# Sensitive B-cell receptor repertoire analysis shows repopulation correlates with clinical response to rituximab in rheumatoid arthritis

**DOI:** 10.1186/s13075-024-03297-7

**Published:** 2024-03-16

**Authors:** Sabrina Pollastro, Anne Musters, Giulia Balzaretti, Ilse Niewold, Barbera van Schaik, Signe Hässler, Catharina M. Verhoef, Marc Pallardy, Antoine van Kampen, Xavier Mariette, Niek de Vries, on behalf of the ABIRISK Consortium, Natacha Szely, Aude Gleizes, Salima Hacein-Bey Abina, Christophe Richez, Martin Soubrier, Jérome Avouac, Olivier Brocq, Jérémie Sellam, Tom Huizinga, Elizabeth Jury, Jessica Manson, Claudia Mauri, Andrea Matucci

**Affiliations:** 1 Department of Clinical Immunology & Rheumatology | Amsterdam Rheumatology & Immunology Centre (ARC), Amsterdam UMC location AMC, University of Amsterdam, Meibergdreef 9, 1105AZ Amsterdam, The Netherlands; 2 Department of Experimental Immunology, Amsterdam Infection & Immunity Institute | Program Inflammatory Diseases, Amsterdam UMC location AMC, University of Amsterdam, Amsterdam, The Netherlands; 3 Epidemiology and Data Science, Amsterdam Public Health research institute, Amsterdam UMC location AMC, University of Amsterdam, Amsterdam, The Netherlands; 4 Université Paris Saclay, INSERM U1018, CESP, Villejuif, France; 5 INSERM UMR 959, Immunology-Immunopathology-Immunotherapy (i3), Sorbonne Université, Paris, France; 6 Publique Hôpitaux de Paris, Hôpital Pitié Salpêtrière, Biotherapy (CIC-BTi), Paris, France; 7 Department of Rheumatology, FlevoHospital, Almere, the Netherlands; 8 Université Paris-Saclay, INSERM, Inflammation, Microbiome, Immunosurveillance, Châtenay-Malabry, France; 9 Université Paris-Saclay, INSERM UMR1184: Center for immunology of viral infections and autoimmune diseases, Le Kremlin Bicêtre, France; 10 Department of Rheumatology, Assistance Publique-Hôpitaux de Paris (AP-HP), Hôpital Bicêtre, Le Kremlin Bicêtre, France

**Keywords:** Rheumatoid arthritis, Rituximab, B cells, B-cell receptor repertoire, AIRR-seq

## Abstract

**Background:**

Although B-cell depleting therapy in rheumatoid arthritis (RA) is clearly effective, response is variable and does not correlate with B cell depletion itself.

**Methods:**

The B-cell receptor (BCR) repertoire was prospectively analyzed in peripheral blood samples of twenty-eight RA patients undergoing rituximab therapy. Timepoints of achieved BCR-depletion and -repopulation were defined based on the percentage of unmutated BCRs in the repertoire. The predictive value of early BCR-depletion (within one-month post-treatment) and early BCR-repopulation (within 6 months post-treatment) on clinical response was assessed.

**Results:**

We observed changes in the peripheral blood BCR repertoire after rituximab treatment, i.e., increased clonal expansion, decreased clonal diversification and increased mutation load which persisted up to 12 months after treatment, but started to revert at month 6. Early BCR depletion was not associated with early clinical response but late depleters did show early response. Patients with early repopulation with unmutated BCRs showed a significant decrease in disease activity in the interval 6 to 12 months. Development of anti-drug antibodies non-significantly correlated with more BCR repopulation.

**Conclusion:**

Our findings indicate that rather than BCR-depletion it is repopulation with unmutated BCRs, possibly from naïve B cells, which induces remission. This suggests that (pre-existing) differences in B-cell turnover between patients explain the interindividual differences in early clinical effect.

**Supplementary Information:**

The online version contains supplementary material available at 10.1186/s13075-024-03297-7.

## Background

The introduction of the B-cell depleting agent rituximab constituted a major revolution in the treatment of autoimmune diseases, which renewed interest in the role of B cells in autoimmunity. In Rheumatoid Arthritis (RA), an autoimmune disease that affects the peripheral joints, pathological studies show a prominent role for B cells in at least a subset of patients. A larger influx of B cells in the synovial tissue has been associated with autoantibody positivity, but also with more radiographic disease progression [1]. This suggests that treatment efficacy might be tightly linked to the contribution of the B-cell compartment in disease pathogenesis.

Although rituximab is clearly effective in certain autoimmune diseases, on the individual level clinical response may vary and is difficult to predict. One of the factors thought to contribute to this variability is the timing and depth of B cell depletion, which has been shown to be very patient specific [2, 3]. However, the extent of B-cell depletion does not correlate evidently with clinical response [4]. This might be due to the fact that our tools are relatively insensitive in monitoring B-cell levels in depleted patients, thus detecting repopulation of B cells too late to prevent disease relapse. And in fact, earlier studies confirmed that in rituximab-treated patients high sensitivity techniques are needed to successfully detect B cell signals when conventional flow cytometry fails [2, 3]. Hence, there is a clear need for a more sensitive, quantitative diagnostic tool that is able to spot B-cell repopulation very early. If indeed this shows a link with disease progression, this might guide clinicians to adapt therapy accordingly.

In a new prospective cohort of RA patients undergoing rituximab therapy, we used adaptive immune receptor repertoire (AIRR) sequencing to analyze clonal dynamics. We confirmed previously reported effects of rituximab treatment on the peripheral blood B-cell receptor (BCR) repertoire. Furthermore, using BCR repertoire analysis we were able to find a link between BCR repopulation and clinical efficacy, thus shedding more light on the mechanism behind rituximab efficacy in RA.

## Methods

### Patients and samples

Thirty-one patients previously diagnosed with RA according to the 2010 ACR/EULAR criteria [5], who were about to start with rituximab treatment were included in the ABIRISK consortium multicentric clinical study (NCT02116504) whose primary objective was to assess predictive factors of anti-drug antibodies (ADA) development (www.abirisk.eu/) [6]. The treatment protocol consisted of two intravenous injections of 1000 mg rituximab (Roche, Woerden, The Netherlands) 15 days apart. A second cycle of treatment was allowed after 6 months at the treating physician’s discretion (*n* = 6). Concomitant medications allowed for RA treatment were Disease-modifying antirheumatic drugs (DMARDs), Non-steroidal anti-inflammatory drugs (NSAID) and corticosteroids. No other biologicals were allowed. Patient visits were at baseline and at one, three, six and twelve months after treatment for sample collection and assessment of disease activity using the Disease Activity Score 28 joints (DAS28) based on CRP, or ESR when CRP was not available. Clinical response was assessed using EULAR response criteria [7].

Peripheral blood for BCR repertoire analysis was collected using PAXGene Blood RNA tubes (PreAnalytiX, Breda, The Netherlands) and stored at -80°C. Serum for Anti-Drug Antibodies (ADA) testing was collected in BD SST vacutainers, left to coagulate for at least 30 min, centrifuged at 1,500 g for 10 min at 4°C and then stored at -20°C.

The study protocol received ethical approval in all patient recruiting centers and was performed according to the Declaration of Helsinki. All patients gave written informed consent before participation.

### Next-generation sequencing of the B-cell receptor repertoire

RNA extraction was performed using PAXgene isolation kit (Qiagen) according to manufacturer’s instructions. Amplification of the B-cell receptor repertoire was performed as previously described and reported in online Supplementary Figure S1A [8, 9]. In case no amplification product was obtained, the amplification was repeated with the addition of carrier RNA from the non BCR-expressing cell line HEK939T. This addition did not alter the sample’s BCR repertoire (online Supplementary Figure S1B-C).

### Processing of raw sequences and final dataset construction

Reads were processed using pRESTO [10]. Low quality reads (phred score ≤ 25) were filtered out. IGHV and IGHJ primer sequences were masked and cut off respectively using the *MaskPrimers.py* function, UMI-based consensus sequences created using *BuildConsensus.py* (max.error = 0.1) and paired-end reads assembled. Unique UMI-based consensus sequences represented by at least 3 different UMIs were aligned using IMGT/HighV-QUEST [11]. Functional rearrangements were further processed for germline reconstruction and clonal clustering using Change-O [12].

### B-cell receptor repertoire analysis

B-cell receptor (BCR) clonotypes were defined as unique IGHV-IGHD-IGHJ rearrangement at the nucleotide sequence level . Abundance was defined as the number of different UMIs associated with each clonotype, expressed as the percentage of total number of UMIs in the sample. Clonal expansion was calculated as the Gini index on the distribution of the number of unique UMIs per BCR clonotype in each sample, and Clonal diversity as the Shannon index on the distribution of the number of unique BCR clonotypes per clonal lineage in each sample [13]. These indices were calculated using the *renyi* function in the vegan R package [14] (version 2.5-6). Analysis of somatic hypermutation was performed using the SHazaM [12] R package (version 0.2.1).

### ADA testing

Serum was tested for presence of ADA against rituximab performing a chemioluminescence drug-tolerant capture ELISA assay using a Meso Scale Diagnostic platform at the clinical immunology laboratory of GlaxoSmithKline Research and Development, Upper Merion, PA, USA.

### Dealing with missing data

Three patients were excluded from the final analysis because the baseline PAXGene sample was not collected. For the remaining 28 patients (baseline characteristics in online Supplementary Table S1), 5 follow-up samples (out of total 112) were not collected and 14 failed BCR amplification or post-sequencing quality control (online Supplementary Table S2). For analysis of response prediction all patients were included based on the assumption that sample failure represented complete B cell depletion. These samples will be later referred to as *imputed* data. This assumption is supported by the fact that available samples taken at the earlier or later timepoint indeed did show extensive BCR depletion (see Fig. 2B). No change in results was observed if these patients were, or were not included (Figs. 3 and 4 and online Supplementary Figure S3). In case of missing DAS28-score (5% of the data), the timepoints concerned were excluded from the analysis. For the analysis of clonality, somatic hypermutation, BCR depletion and BCR repopulation after rituximab, patients were included if they had no (*n* = 18) or one (*n* = 5) missing follow-up timepoint (total *n* = 23).

### Statistics

Data are presented as mean and standard deviation (SD) or median and interquartile range (IQR) after D’Agostino and Pearson omnibus test for normality. Differences between groups were evaluated using unpaired t-test and one-way analysis of variance (ANOVA) followed by Bonferroni’s multiple comparisons post-test for normally distributed data or Mann-Whitney test and Kruskal-Wallis test followed by Dunn’s multiple comparisons post-test for not-normally distributed data. Contingency tables were evaluated with Fisher's exact test. *P*-values less than 0.05 were considered statistically significant. Graphpad Prism 7 software (Graph Pad, San Diego, CA, USA) was used to perform the statistical tests.

## Results

### The impact of rituximab on the B cell receptor repertoire persists for up to one year

It has been shown that 4 weeks after treatment with rituximab the peripheral blood BCR repertoire is characterized by more expanded and mutated BCR clonotypes [3]. To confirm and extend these findings, we prospectively analyzed a cohort of 23 (see Methods section ‘ Dealing with missing data’) rituximab-treated RA patients with one year follow-up after treatment.

**Fig. 1:**
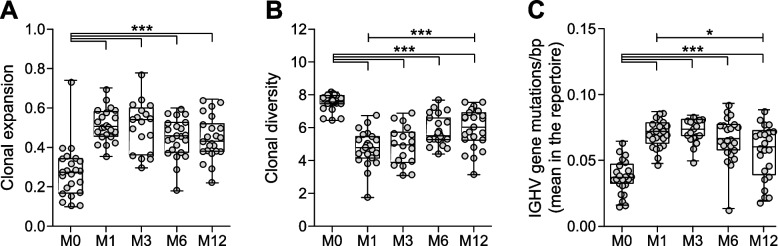
*Peripheral blood BCR repertoire in patients undergoing rituximab treatment*. Boxplots showing (**A**) clonal expansion: Gini index, (**B**) clonal diversity: Shannon index and (**C**) the average IGHV gene mutation load in samples obtained before (M0), and at one (M1), three (M3), six (M6) and twelve (M12) months after treatment with rituximab. Boxplots show the median, 25th and 75th interquartile, error bars show the range, and single data points are depicted in grey (*p ≤ 0.05, ***p ≤ 0.001, using one-way ANOVA)

At one month after treatment, B cells were undetectable in all patients using classical cytometry. Using AIRR, at one month clonal expansion was significantly higher (*p* < 0.001; 0.52 ± 0.08 vs. 0.27 ± 0.14, mean ± SD; Fig. 1A), while clonal diversity was significantly lower compared to baseline (*p* < 0.001; 4.75 ± 1.06 vs. 7.53 ± 0.49, mean ± SD; Fig. 1B), as expected after B-cell depleting therapy. In addition, BCR clonotypes carried a significantly higher number of somatic hypermutations in the IGHV gene (*p* < 0.001; mutations/bp 0.07 ± 0.01 vs. 0.04± 0.01, mean ± SD; Fig. 1C). These data confirm that one month after treatment with rituximab the BCR repertoire is dominated by fewer, more expanded and more mutated BCR clonotypes.

Further analysis at the follow-up timepoints revealed a trend towards re-establishment of pre-treatment conditions from month 6 post-treatment onwards for all three analyzed indexes. Compared to month 1, the BCR repertoire at month 12 showed significantly higher clonal diversity (5.93 ± 1.20; *p* < 0.001) and less IGHV gene mutations (0.06 ± 0.02; *p* < 0.05). Of note, this effect was even more accentuated when excluding re-treated patients at month 6 (online Supplementary Figure S2). Taken together these results show that rituximab induces profound changes on the peripheral blood BCR clonality. In some patients these changes persist for up to 12 months after treatment while in others restoration of the pre-treatment conditions starts at month 6 after treatment.

### Defining BCR-depletion and BCR-repopulation timepoint

The trend to re-establish pre-treatment conditions, observed in the peripheral blood BCR repertoire 6 months post-treatment, might reflect the restoration of the B-cell compartment after B-cell depletion. It has been reported that repopulation of B cells after depletion starts with antigen naïve B cells with reduced somatic hypermutation in the IGHV genes [15, 16]. BCR repertoire analysis in different B cell subsets from healthy donors showed that such naïve B cell repertoires mostly contain unmutated BCR sequences [13, 17]. We therefore speculated that the percentage of unmutated BCR clonotypes in the total BCR repertoire could be used as a proxy for the presence of antigen-naïve B cells among the BCR-expressing cells in peripheral blood. A decreasing percentage would indicate that rituximab is depleting these cells from the circulation; an increasing percentage would indicate that the influx of these cells in peripheral blood is restored, as observed during repopulation.

**Fig. 2:**
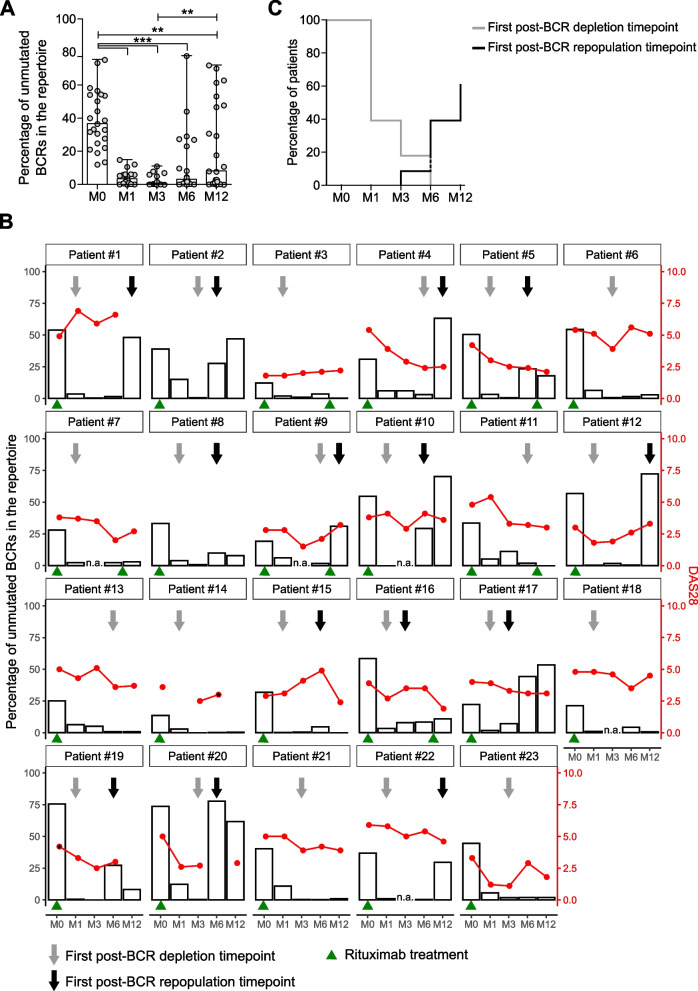
*Monitoring of changes in the peripheral blood BCR repertoire during treatment with rituximab*. **A** Barplot showing the percentage of unmutated clonotypes in samples obtained before (M0), and at one (M1), three (M3), six (M6) and twelve (M12) months after treatment with rituximab. Bars height shows the median, error bars show the range. Single data points are depicted in grey (**p ≤ 0.01, ***p ≤ 0.001, using one-way ANOVA). **B** Disease activity score (DAS28: red dots and line, red scale on the right Y-axis) and percentage of unmutated clonotypes (white bars, scale on the left Y-axis) in the individual patients. Grey arrows indicate the first post-BCR depletion timepoint, while black arrows indicate the first post-BCR repopulation timepoint. Green triangles indicate the timepoint at which each patient received rituximab treatment. **C** Percentage of patients that reached the first post-BCR depletion (grey) and first post-BCR repopulation timepoint (black) at the timepoints analyzed

The percentage of unmutated BCR sequences in the total repertoire significantly decreased one month after treatment compared to baseline (p ≤ 0.001; 4.33 ± 3.98 vs. 40.0 ± 17.8, mean ± SD, Fig. 2A). This decrease was at its lowest at month 3, and persisted up to month 12. Of note, when we excluded re-treated patients, the percentage of unmutated BCRs at 12 months did not differ significantly from that at baseline (online Supplementary Figure S2D). From month 6 onwards we observed an increase in the percentage of unmutated BCR sequences, which at month 12 was significantly different compared to the dip at month 3 (p ≤ 0.01; 23.1 ± 26.5 vs. 2.54 ± 3.38, mean ± SD). Overall, it was clear that the observed decrease and increase in the percentage of unmutated BCRs in the repertoire was patient specific.

To address this variability, we defined for each patient a *post-BCR depletion* and a *post-BCR repopulation* timepoint as the first timepoint at which the percentage of unmutated BCRs in the repertoire respectively drops below and increases again above 4.4% (mean minus two-times the standard deviation of the unmutated BCRs distribution at baseline) (Fig. 2B, grey and black arrow). Using these definitions, we observed that out of the 23 patients, 14 (61%) reached their *post-BCR depletion* endpoint at month 1, 19 (83%) at month 3, and 23 (100%) at month 6 after treatment (Fig. 2C, grey line). Regarding B cell repopulation, cumulatively 2 (9%) patients reached their *post-BCR repopulation* endpoint at month 3, 9 (39%) at month 6, and 14 (61%) at month 12 after treatment (Fig. 2C, black line). In the remaining 9 (39%) patients no *post-BCR repopulation* timepoint could be defined within the follow-up period of 1 year. In conclusion, using this novel approach our data confirm that B-cell depletion is achieved within one month of treatment in the majority of the patients, and show that B cell repopulation takes more than 12 months in one third of the treated patients.

### No correlation of BCR depletion and treatment outcome

**Fig. 3:**
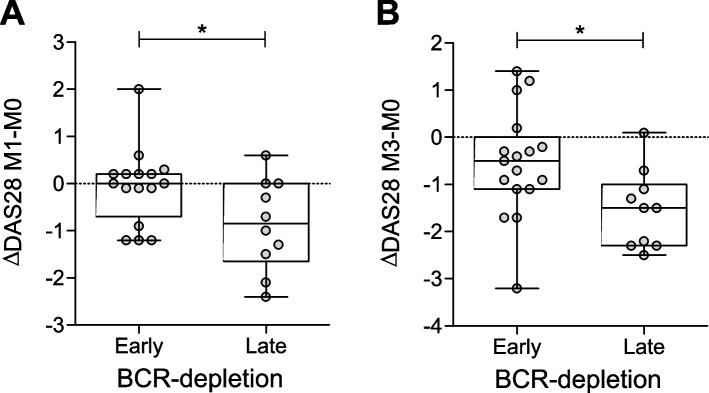
*Timing of BCR depletion does not predict long-term clinical outcome but correlates with short-term disease activity*. **A**-**B** Boxplot showing the changes in DAS28-score between month 1 (**A**) or month 3 (**B**) post-treatment and baseline in early or late depleting patients. Boxplots show the median and 25th and 75th interquartile, error bars show the range, and single data points are depicted in grey (*p ≤ 0.05, using Mann-Whitney test in A and unpaired t-test in B)

To answer the question whether timing of BCR depletion correlates with treatment efficacy, we evaluated changes in DAS28 and clinical response separately in patients who had their first *post-BCR depletion* timepoint within one month after treatment (early BCR-depleting group), and patients who achieved this endpoint later during the follow-up (late BCR-depleting group). Interestingly, while achieving the post-BCR depletion timepoint at one month, patients in the early depleting group did not show any change in DAS28 at this timepoint. In contrast, late depleting patients did show a decrease in DAS28-score one-month post-treatment, significantly different from that in early depleting patients (median (IQR): -0.9 (-1.7 – 0.0) vs. 0.0 (-0.7 – 0.2); *p*-value = 0.04); Fig. 3A and online Supplementary Figure S3A excluding imputed data). This effect was still observed after 3 months (-1.5 ± 0.8 vs. -0.50 ± 1.1; *p*-value = 0.02; Fig. 3B and online Supplementary Figure S3B). At month 6 or 12 the groups did not show significant differences in changes in DAS28 and in clinical response (data not shown). Taken together these data indicate that timing of BCR depletion is not correlated to long-term therapy response, and suggest that a delayed achievement of BCR depletion after treatment might be associated with reduced disease activity in the first three months.

### Correlation of BCR repopulation with treatment outcome

**Fig. 4:**
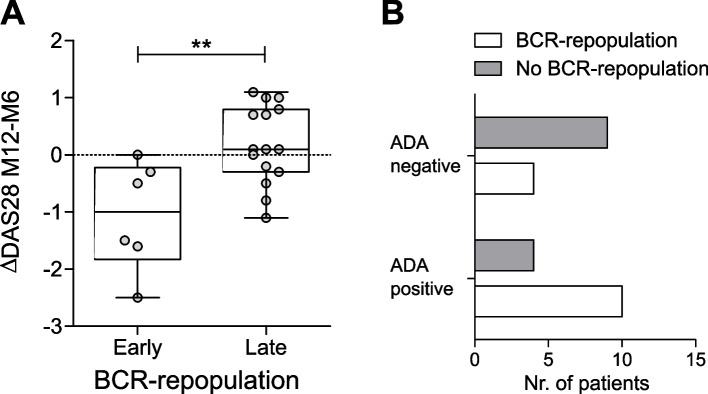
*BCR repopulation is associated with improvement of disease activity shortly after and possibly with anti-drug antibodies development*. **A** Boxplot showing the changes in DAS28-score between the month 6 and month 12 after treatment in early or late  BCR repopulating patients. Boxplots show the median and 25th and 75th interquartile, error bars show the range, and single data points are depicted in grey (** p ≤ 0.01, using unpaired t-test). **B** Barplot showing the correlation between anti-drugs antibodies (ADA) development and repopulation within 12 months of treatment

Next, we evaluated whether the timing of repopulation of the B-cell compartment is correlated with treatment outcome. Patients were divided into an “early” or “late” BCR repopulation group, with early repopulating patients having their first *post-BCR repopulation* timepoint within 6 months after treatment. Compared to baseline, no difference in DAS28 changes, or in clinical response at month 6 or 12 post-treatment was observed between the two groups (data not shown). However, there was a significant difference when analyzing the change in DAS28-score after repopulation (Fig. 4A). In the early repopulating patients, a larger decrease in DAS28-score was measured between month 6 and 12 (*p*-value < 0.01; early repopulating: -1.17 ± 0.96, late repopulating: 0.18 ± 0.67, mean ± SD). This was also observed when excluding re-treated patients (online Supplementary Figure S2E) or excluding imputed data (online Supplementary Figure S3C). Of note, the DAS28 at month 6 did not differ between the two groups (early repopulating: 3.36 ± 0.89, late repopulating: 3.42 ± 1.53, mean ± SD; *p*-value = 0.92), thus not explaining this observation. In addition, there was no correlation between early BCR depletion and early BCR repopulation (*p*-value = 0.41), indicating that early BCR depletion does not lead to early repopulation. Thus, early repopulation of the B cell compartment does not predict treatment outcome at 6 or 12 months, but is associated with improvement of disease activity shortly after repopulation.

### Effect of anti-drug antibodies on BCR repopulation

ADAs are known to increase drug clearance due to the formation of ADA-drug complexes, and patients who develop ADAs show a reduced drug trough concentration in serum [18, 19]. We therefore assessed whether development of ADAs against rituximab was associated with early B cell repopulation. In our cohort of 28 patients, 14 developed ADAs within 12 months of treatment. No association between ADA development and BCR depletion (early or late) was observed (data not shown). Ten (71%) out of these 14 ADA positive patients showed B-cell repopulation within 12 months, while 4 out of 13 (30%) of the ADA negative patients did (*p*-value = 0.06, n.s., 1-sided Fisher exact test; Fig. 4B and online Supplementary Figure S3D. For one patient no repopulation status was available). Hence, even though not significant, there still might be a trend in favor of an association between the development of anti-drugs antibodies within 12 months of treatment and repopulation of the B cell compartment within the same time-span.

## Discussion

Using UMI-based adaptive immune receptor repertoire (AIRR) sequencing in RA patients undergoing rituximab treatment we show that deletion and recurrence of the unmutated BCRs, associated with the naive repertoire, proved a sensitive marker for depletion and repopulation. Using this parameter, we find a correlation between BCR repopulation and clinical improvement shortly after, strongly suggesting that it might be the repopulation, rather than the depletion, that has a dampening role on disease activity.

As described earlier, treatment with rituximab leads to fewer, more expanded and more mutated BCR clonotypes [3]. This is not surprising since the residual B cell population is most likely composed of plasmablasts and plasma cells which do not express CD20, the molecular target of rituximab. Therefore, these cells are not affected by this B cell depleting therapy. In other studies, phenotypic characterization of the post-depletion B cell compartment using highly sensitive flow cytometry showed the presence of a residual memory B cell population which was also the predominant re-populating fraction in patients with early relapse after B cell recovery [2, 15, 16]. Even though our RNA-based analysis is dominated by the BCR signal coming from plasmablasts/plasma cells due to their higher RNA content (data not shown from our lab), we cannot exclude that some of the residual dominant BCR clones observed in our analysis still represent memory B cells.

In this study we monitored unmutated clonotypes in the BCR repertoire with quantitative UMI-based sequencing to sensitively follow the dynamics of B cell depletion and repopulation in rituximab treated patients. Using this method, we observed that *repopulation* of the BCR repertoire within 6 months of treatment did correlate with significantly better disease amelioration in the subsequent period, between month 6 and 12 post treatment, compared to patients that did achieve repopulation later or not at all (*p*-value < 0.01). This observation was not explained by a difference in disease activity at 6 months between the two groups (*p*-value = 0.92) nor by a 2^nd^ cycle of treatment. This might indicate that it is in fact the repopulation following rituximab – rather than depletion itself - that is able to “re-set" the (pathological) B cell compartment, leading to temporal improvement of the disease activity. In this context, the ability to promptly recognize the start of B cell repopulation after depletion might thus be extremely useful in clinical practice, allowing the physicians to more closely monitor disease progression during repopulation and eventually take an informed decision on intensification of treatment.

Using the same approach, we observed that BCR *depletion* does not predict clinical response at 6 and 12 months. On top of that, we showed that patients that achieve BCR depletion within one month of treatment did not show any improvement in disease activity in the same time-span. This confirms previous reports showing that timing and depth of B cell depletion does not correlate with clinical response to rituximab during 0.5 and 1 year of follow-up [4, 20]. This lack of correlation is thus not due to a lack of sensitivity of previously used methods. The observed association of early BCR repertoire depletion with *less* response at 1 and 3 months after rituximab therapy is interesting. This might be the result of the fact that in our cohort late depleting patients had a slightly higher baseline DAS28 score (late depleting: 4.6 ± 0.9 vs. early depleting: 4.1 ± 1.1; *p*-value = 0.2, n.s.), thus resulting in more potential to improve in disease activity [20, 21]. However, if indeed an influx of naïve, unmutated B cells explains the clinical effect of rituximab, an alternative explanation might be that part of the patients have a higher baseline influx of naïve, unmutated B-cells before treatment; this increase in B-cell turnover might explain both later BCR depletion with better amelioration of disease activity, and earlier BCR repopulation with better disease amelioration. In case of rituximab, this would lead to a complicated relation between anti-drug antibodies (ADA) development and clinical response. In patients newly starting on rituximab, some will develop ADA after 3 to 6 months [6]. In these patients, ADA is likely to be correlated with earlier clearance of the drug and earlier repopulation (*p* = 0.06 in our data) leading to better clinical response. However, in patients with pre-existing ADA, these antibodies will interfere with the primary effect of rituximab, i.e. B-cell depletion, and therefore lead to clinical non-response. In this situation the net effect of both mechanisms will determine the clinical response in each individual patient. 

In this study we showed how our BCR-based approach could be adopted by clinician to monitor the dynamics of BCR-depletion and -repopulation during treatment with rituximab. Yet, there are several points which still need to be addressed before our approach can reach clinical practice. In first instance, this study needs be repeated in additional cohorts. This would also allow to define a more robust cut-off for BCR-depletion and BCR-repopulation. Secondly, this approach should be compared with other routinely used methodologies for the sensitive quantification of B cells during B cell depletion therapy, such as high-sensitivity flow cytometry and DNA-based NGS sequencing of immunoglobulin gene rearrangements [2, 22]. In this respect, the use of a DNA-based rather than RNA-based approach would allow for a more precise quantification of the number of B cells rather than the number of B-cell receptor sequences without the need for a flow-based quantification. Yet the difficulty in incorporating unique molecular identifiers (UMI) in this approach makes it difficult to correct for primer-based amplification bias. Finally, to get more insight in the underlying biology, it would be interesting to further explore the phenotypic diversity of B cells carrying unmutated BCRs and the impact of these B cells on the reported correlations with clinical response.

The protocol adopted in this study has several limitations. One is the relatively short follow-up time. Since most of the patients show repopulation of the BCR repertoire at 6 months post-treatment, having the last follow-up point set at 12 months post treatment was relatively short to detect disease relapse. The second limitation is that the protocol did allow intensification of treatment in cases of insufficient response. However, clinicians did not have access to the results of the BCR repopulation analysis, and the decision to retreat with rituximab after 6 months did not correlate with early repopulation (*p* = 0.7). It would have been nice to correlate the BCR repertoire in paired synovial tissue samples with that in peripheral blood to study the recurrence of B cell clonotypes during disease relapse. Such a study could prove that rituximab does not eradicate pathological B cells but just prevents them to reach the site of disease activity, i.e., the synovium, therefore explaining why - despite the temporary amelioration of disease symptoms - CD20-depleting therapy does not cure RA.

## Conclusions

Using AIRR-sequencing to monitor the dynamics of BCR repertoire after rituximab treatment we observed that timing of BCR depletion and BCR repopulation does not predict response after 6 or 12 months. However, BCR repopulation within 6 months did significantly correlate with response in the subsequent interval (6-12 months), suggesting it is repopulation rather than depletion that influences long-term remission. Based on our results, we hypothesize that interindividual differences in clinical response to rituximab are defined by patient-specific differences in B-cell turnover. We propose further studies are indicated to validate the latter observations, analyze the underlying mechanisms and assess which cell populations are involved.

### Supplementary Information

**Supplementary Material 1.** 

## Data Availability

Raw sequencing reads have been deposited at NCBI Sequence Read Archive (BioProject: PRJNA943359) and processed repertoires and code used are available upon request to the corresponding author.

## Abbreviations

RA: Rheumatoid arthritis
BCR: B-cell receptor
AIRR: Adaptive immune receptor repertoire
ACR: American College of Rheumatology
EULAR: European Alliance of Associations for Rheumatology
ADA: Anti-drug antibodies
DAS28: Disease Activity Score 28 joints
DMARDs: Disease-modifying antirheumatic drugs
NSAID: Non-steroidal anti-inflammatory drugs
CRP: C-reactive protein
ESR: Erythrocyte sedimentation rate
IGHV: Immunoglobulin heavy-chain variable region
IGHD: Immunoglobulin heavy-chain diversity region
IGHJ: Immunoglobulin heavy-chain joining region
UMI: Unique molecular identifier
ELISA: Enzyme-linked immunosorbent assay
SD: Standard deviation
IQR: Interquartile range
ANOVA: One-way analysis of variance
