# Supplementary material for: Sensitive B-cell receptor repertoire analysis shows repopulation correlates with clinical response to rituximab in rheumatoid arthritis
Source: Arthritis Res Ther. 2024 Mar 16;26:70. doi: 10.1186/s13075-024-03297-7 (PMC10943808; doi:10.1186/s13075-024-03297-7)
Supplement: Supplementary file 1 — Supplementary Material 1. [file 13075_2024_3297_MOESM1_ESM.pdf]

Sensitive B-cell receptor repertoire analysis shows repopulation correlates with clinical response to rituximab in rheumatoid arthritis

Sabrina Pollastro[1,2†], Anne Musters[1†], Giulia Balzaretti[1,2†], Ilse Niewold[1], Barbera van Schaik[3], Signe Hässler[4,5,6], Catharina M. Verhoef[7], Marc Pallardy[8], Antoine van Kampen[3], Xavier Mariette[9,10], Niek de Vries[1,2*], on behalf of the ABIRISK Consortium.

[1]Department of Clinical Immunology & Rheumatology | Amsterdam Rheumatology & Immunology Centre (ARC), Amsterdam UMC location AMC, University of Amsterdam; Amsterdam, The Netherlands.
[2]Department of Experimental Immunology | Amsterdam Infection & Immunity Institute | Program Inflammatory Diseases, Amsterdam UMC location AMC, University of Amsterdam; Amsterdam, The Netherlands.
[3]Epidemiology and Data Science, Amsterdam Public Health research institute, Amsterdam UMC location AMC, University of Amsterdam; Amsterdam, The Netherlands.
[4]Université Paris Saclay, INSERM U1018, CESP; Villejuif, France.
[5]INSERM UMR 959, Immunology-Immunopathology-Immunotherapy (i3), Sorbonne Université; Paris, France.
[6]Assistance Publique Hôpitaux de Paris, Hôpital Pitié Salpêtrière, Biotherapy (CIC-BTi); Paris, France.
[7]FlevoHospital, Department of Rheumatology; Almere, the Netherlands,
[8]Université Paris-Saclay, INSERM, Inflammation, Microbiome, Immunosurveillance; Châtenay-Malabry, France.
[9] Université Paris-Saclay, INSERM UMR1184: Center for immunology of viral infections and autoimmune diseases; Le Kremlin Bicêtre, France.
[10]Department of Rheumatology, Assistance Publique-Hôpitaux de Paris (AP-HP), Hôpital Bicêtre; Le Kremlin Bicêtre, France.

SUPPLEMENTARY MATERIAL

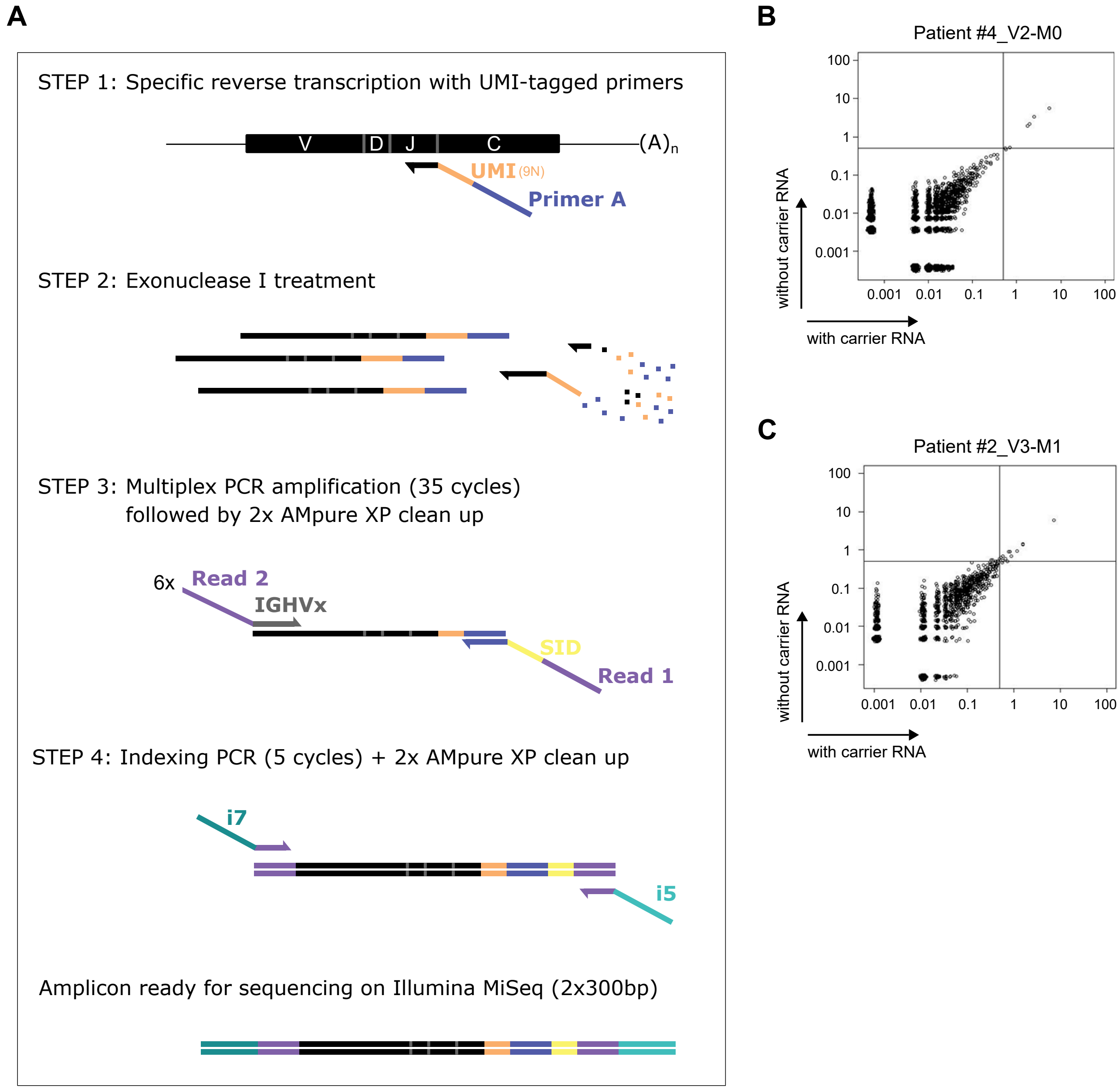

Figure S1 | New UMI-based BCR repertoire sequencing pipeline.
A) Schematic representation of the high-throughput UMI-based BCR repertoire sequencing workflow. Specific reverse transcription of BCRh RNA molecules is performed with UMI-tagged primers complementary to the BCR heavy chain joining gene (step 1). Exonuclease I treatment is performed to remove unbound reverse transcription primers (step 2) before performing a multiplexed PCR with 6 BCRh variable (IGHV) chain forward primers and a single reverse primer (step 3). Obtained amplicons are then indexed with i7 and i5 Nextera Illumina indexes (step 4) and sequenced on an Illumina MiSeq platform.
B-C) Scatter plots depicting the clonal overlap in the same pre-treatment (B) or post-treatment (C) sample amplified with or without addition of carrier RNA from the non BCR-expressing cell line HEK939T prior cDNA synthesis. X- and Y-axes depict the frequency of each BCR clonotype in the repertoire as percentage of total UMIs.

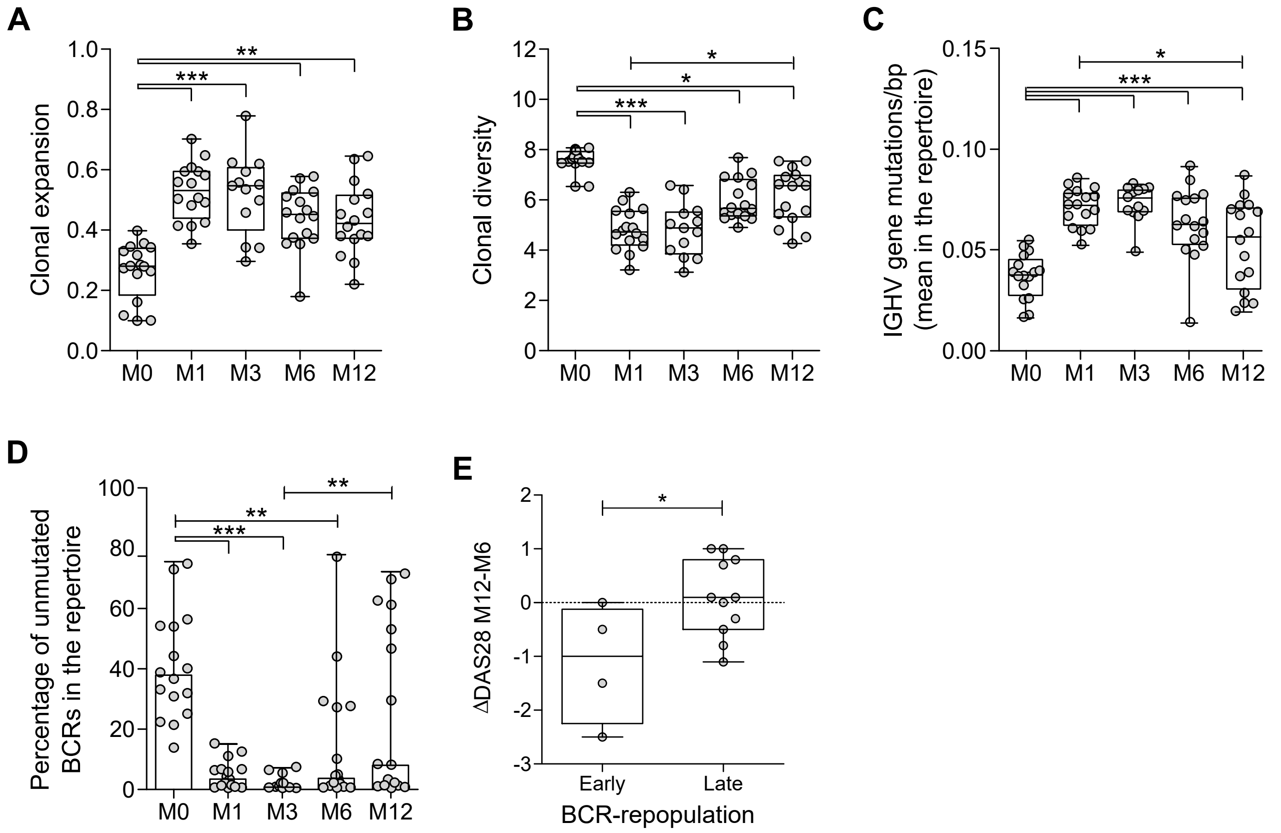

Figure S2 | Results excluding patients who received a 2nd cycle of treatment (n=6).
A-C) Boxplots showing (A) the clonal expansion: Gini index, (B) the clonal diversity: Shannon index and (C) the average IGHV gene mutation load in samples obtained before (M0), and at one (M1), three (M3), six (M6) and twelve (M12) months after treatment with rituximab. Boxplots show the median, 25% and 75% interquartile range and error bars show the range. Single data points are depicted in grey. D) Barplot showing the percentage of unmutated clonotypes before (M0), and at one (M1), three (M3), six (M6) and twelve (M12) months after treatment with rituximab. Bars height shows the median, error bars show the range. Single data points are depicted in grey. E) Boxplot showing the changes in DAS28-score between the month 6 and month 12 after treatment in early or late repopulating patients. Boxplots show the median and 25th and 75th interquartile and error bars show the range. Single data points are depicted in grey.
(*p ≤ 0.05, **p ≤ 0.01, ***p ≤ 0.001, using Kruskal-Wallis test in A and B, one-way ANOVA in C and D and unpaired t-test in E).

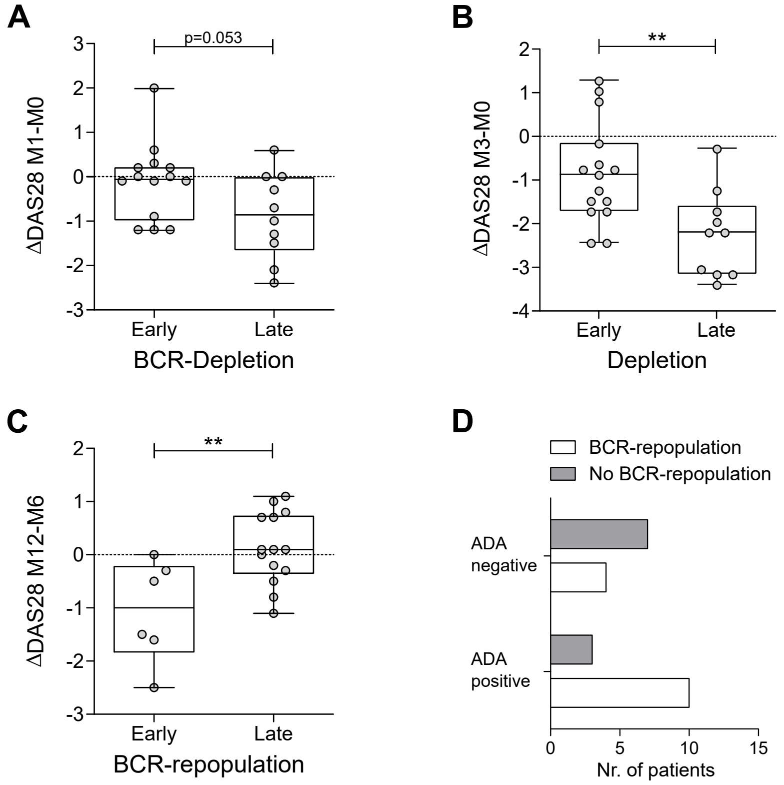

Figure S3 | Results excluding imputed data on depletion and repopulation.
A-C) Boxplot showing the changes in DAS28-score between month 1 (A) or month 3 (B) post-treatment and baseline in early or late depleting patients and (C) the changes in DAS28-score between the month 6 and month 12 after treatment in early or late repopulating patients. Boxplots show the median and 25th and 75th interquartile and error bars show the range. Single data points are depicted in grey (** $p \leq 0.01$, using unpaired t-test in B and C). D) Barplot showing the correlation between anti-drugs antibodies (ADA) development and repopulation within 12 months of treatment.

Table S1 | Patients baseline characteristics**.**

**Demographics**	n=28
Median age, years (range)	61 (21-79)
Female, no. (%)	22 (79)
Never smoker, no (%)	12 (43)
**Disease status**	
Median disease duration, years (range)	12 (1-28)
IgM-RF positive, no. (%)*	20 (74)
ACPA positive, no. (%)**	20 (80)
Median DAS28 (IQR)*	4.2 (2.6-6.8)
Median CRP, mg/L (IQR)*	5.2 (1.2-109)

IgM-RF, IgM-rheumatoid factor; ACPA, anti-citrullinated protein antibodies; ESR, erythrocyte sedimentation rate; CRP, C-reactive protein. *one missing data (n=27); ** three missing data (n=25)

Table S2 | List of analyzed samples

Patient	Timepoint: Baseline (M0)	Month 1 (M1)	Month 3 (M3)	Month 6 (M6)	Month 12 (M12)
# 1					
# 2					
# 3					
# 4					
# 5					
# 6					
# 7			[gray]		
# 8					
# 9					
# 10					
# 11					
# 12					
# 13					
# 14			[gray]		
# 15			[gray]		
# 16					
# 17					
# 18					
# 19			[gray]		
# 20					
# 21					
# 22			[black]		
# 23					
# 24		[gray]	[gray]	[gray]	[gray]
# 25			[black]	[black]	[black]
# 26			[gray]	[gray]	
# 27		[gray]	[gray]		[gray]
# 28			[black]		[gray]

Black: not collected; Gray: BCR failed
